# Data on the biomass of commercially important coral reef fishes inside and outside marine protected areas in the Philippines

**DOI:** 10.1016/j.dib.2019.104176

**Published:** 2019-06-25

**Authors:** Richard N. Muallil, Melchor R. Deocadez, Renmar Jun S. Martinez, Porfirio M. Aliño

**Affiliations:** a Mindanao State University – Tawi-Tawi College of Technology and Oceanography, Bongao, Tawi-Tawi 7500, Philippines; b Marine Environment and Resources Foundation, Inc., Marine Science Institute, University of the Philippines Diliman, 1101 Quezon City, Philippines; c Marine Science Institute, University of the Philippines Diliman, 1101 Quezon City, Philippines

**Keywords:** Marine protected area, Coral reef fishes, Sustainable fishery, Fish biomass, Coral triangle

## Abstract

This article contains the data on fish biomass inside and outside 57 locally managed marine protected areas (MPAs) and within the nationally protected Tubbataha Reef National Marine Park (TRNMP) from 57 coastal municipalities and 20 provinces in the Philippines. It includes the seven major commercially important coral reef fishes, namely, the surgeonfish (family Acanthuridae), parrotfish (subfamily Scarinae, family Labridae), snappers (family Lutjanidae), groupers (subfamily Epinephelinae, family Serranidae), goatfish (family Mullidae), sweetlips (family Haemulidae) and emperor (family Lethrinidae). Fish visual census (FVC) surveys were done by scuba diving along 10 m × 50 m belt transects established on upper reef slope, mostly with depths ranging from 5 to 10 m. Four to twelve transects were surveyed for the locally managed MPAs, half of which were established inside MPAs and the other half outside MPAs. Thirty-three transects were surveyed for the TRNMP. FVC was performed by swimming slowly and stopping every 5 m to record all the fish within a 10 m - wide belt. All FVC surveys were conducted from 2006 to 2014 between 9:00–16:00 hours. Each fish was identified to the species level and total length (TL) was estimated to the nearest centimeter. Fish biomass was estimated using the relationship between length (L) and weight (W) with the equation W = aL^b^. The data we provide can be used for coral reef fisheries management and for monitoring and evaluation of coral reef fishes in the Philippines particularly for the MPAs included in this dataset. These data support the information presented in the article Muallil et al., 2019.

**Table:** Specifications table

Subject	Biology
Specific subject area	Biomass of commercially important coral reef fishes inside and outside marine protected areas in the Philippines
Type of data	Table
How data were acquired	Data were collected by fish visual census (FVC) surveys which were done by scuba diving along 10 m × 50 m belt transects established on upper reef slope, mostly with depths ranging from 5 to 10 m. FVC was performed by swimming slowly and stopping every 5 m to record all the fish within a 10 m - wide belt.
Data format	Analyzed
Parameters for data collection	FVC surveys were conducted on belt transects established on upper reef slope, mostly with depths ranging from 5 to 10 m. 4–12 belt transects were surveyed in each site, half of which were established inside MPAs and the other half on adjacent reefs outside MPAs, which were at least 200 m away from the boundaries of MPAs. All surveys were conducted from 2006 to 2014 between 9:00–16:00 hours, mostly when the weather was calm.
Description of data collection	Data were collected using the modified fish visual census (FVC) of English et al. (1997) [2] by scuba diving along 10 m × 50 m belt transects. FVC was performed by swimming slowly and stopping every 5 m to record all the fish within a 10 m - wide belt. Each fish was identified to the species level and total length (TL) was estimated to the nearest centimeter. Fish biomass was estimated using the relationship between length (L) and weight (W) with the equation W = aL^b^. Species specific *a* and *b* values were gathered from published records [3], [4].
Data source location	The data include 57 locally managed MPAs and the nationally managed Tubbataha Reef National Marine Park (TRNMP) from 57 coastal municipalities and 20 provinces in the Philippines.
Data accessibility	With the article
Related research article	Muallil, R.N., Deocadez, M.R., Martinez, R.J.S., Campos, W.L., Mamauag, S.S., Nañola Jr., C.L., Aliño, P.M., 2019. Effectiveness of small locally-managed marine protected areas for coral reef fisheries management in the Philippines. Ocean Coast Manage. 179, 104 831

**Table:** 

**Value of the data** • The Philippines lies within the Coral Triangle which is a major hotspot for coral reef biodiversity because it is the area with the richest coral reef biodiversity but also most threatened from anthropogenic disturbances. • The data can be used by scientists, government agencies (e.g. Bureau of Fisheries and Aquatic Resources (BFAR), Department of Environment and Natural Resources (DENR) and Department of Tourism (DOT), local government units (LGUs), MPA managers and coastal resource management (CRM) practitioners. • The data can be used to derive insight to create policies on coral reef conservation and fisheries management. The data can also be used for monitoring and evaluation purposes. • These data can help in the prioritization of key areas for coral reef monitoring and conservation programs.

## 1 Data

The article includes transect-level (Table 1) and the mean biomass data per site (Table 2) of commercially important coral reef fishes collected from a total of 507 500-m^2^ transects in 58 sites from 57 coastal municipalities/city and 20 provinces covering all the six biogeographic regions of the Philippines [1]. Thirty-three of the transects surveyed were from the nationally protected Tubbataha Reef National Marine Park (TRNMP) while the rest were from the locally managed marine protected areas (MPAs).

**Table 1:** Transect-level fish biomass (mt/km^2^) of commercially important coral reef fishes (by family or subfamily) inside and outside MPAs.

Municipality	MPA Zone	Trasect no.	Acanthurdiae	Haemulidae	Lethrinidae	Lutjanidae	Mullidae	Scarinae	Epinephelinae	Total
San Fernando City	Inside	1	1.1	0.0	0.0	0.3	0.3	0.2	0.9	2.8
	Inside	2	0.5	0.0	0.0	0.0	0.0	0.4	0.0	0.8
	Inside	3	2.0	0.0	0.0	0.0	0.2	0.0	0.8	3.0
	Inside	4	1.4	0.0	0.0	0.4	0.4	0.1	2.8	5.2
	Outside	5	0.5	0.0	0.0	0.0	0.4	0.1	0.6	1.6
	Outside	6	1.7	0.0	0.0	0.0	0.0	0.5	0.3	2.5
	Outside	7	0.9	0.0	0.0	0.0	0.0	0.6	0.2	1.7
	Outside	8	2.1	0.0	0.6	0.0	0.0	1.1	0.1	4.0
Alaminos City	Inside	1	0.0	0.0	0.0	0.0	0.0	0.1	0.1	0.2
	Inside	2	0.8	0.0	0.1	0.4	0.3	1.4	0.2	3.1
	Inside	3	0.1	0.0	0.0	0.1	0.0	0.8	0.4	1.5
	Inside	4	0.4	0.0	0.0	0.1	0.0	2.3	0.2	2.9
	Inside	5	1.3	0.0	0.0	0.3	0.8	1.2	0.1	3.7
	Outside	6	2.3	0.0	0.0	0.0	0.6	5.1	0.4	8.5
	Outside	7	6.5	1.2	0.0	1.3	1.0	3.0	0.1	13.2
	Outside	8	2.1	0.1	0.0	0.0	0.0	0.3	0.2	2.6
	Outside	9	4.5	0.0	0.0	0.3	0.0	0.0	0.0	4.9
	Outside	10	0.5	0.0	0.0	0.0	0.0	0.4	0.0	1.0
Bolinao	Inside	1	5.0	0.0	0.0	2.5	0.1	1.4	1.1	10.1
	Inside	2	5.9	0.4	0.5	0.3	0.3	2.0	0.8	10.2
	Inside	3	5.5	0.3	0.0	0.0	0.3	0.8	0.4	7.3
	Inside	4	0.0	0.0	0.0	0.0	0.0	0.0	0.0	0.0
	Outside	5	7.3	1.0	0.0	0.3	1.1	1.8	0.8	12.4
	Outside	6	1.4	0.0	0.0	0.6	0.2	0.3	0.7	3.1
	Outside	7	6.9	0.0	0.0	0.0	1.3	0.7	0.8	9.6
	Outside	8	0.0	0.0	0.0	0.0	0.0	0.0	0.0	0.0
Candelaria	Inside	1	5.2	0.0	0.0	0.0	0.2	2.2	0.3	7.8
	Inside	2	5.0	0.0	0.0	0.0	1.3	1.4	0.3	8.0
	Inside	3	1.9	0.0	0.0	0.0	0.0	0.6	2.7	5.2
	Inside	4	1.7	0.0	0.0	0.0	0.4	0.2	1.5	3.8
	Inside	5	2.7	0.0	0.0	0.2	1.1	4.5	0.1	8.6
	Inside	6	3.2	0.0	0.0	0.0	0.3	4.3	0.3	8.2
	Outside	7	0.6	0.1	0.0	0.0	0.5	0.6	0.2	2.0
	Outside	8	0.8	0.0	0.0	0.0	0.0	1.7	0.1	2.6
	Outside	9	1.3	0.0	0.0	0.0	0.0	0.0	0.3	1.6
	Outside	10	0.6	0.0	0.0	0.0	0.0	0.0	0.0	0.6
	Outside	1	0.1	0.0	0.0	0.0	0.0	0.0	0.4	0.5
	Outside	2	0.3	0.0	0.0	0.0	0.0	1.0	0.1	1.4
Masinloc	Inside	1	13.3	0.0	0.0	0.2	0.6	1.6	0.0	15.8
	Inside	2	4.6	0.0	0.0	0.5	0.5	1.5	0.4	7.4
	Inside	3	7.2	0.0	0.0	0.0	0.1	2.8	0.1	10.2
	Inside	4	15.0	0.0	1.7	1.5	1.5	4.0	0.3	24.0
	Outside	5	1.8	0.0	0.6	0.0	0.0	0.6	0.3	3.2
	Outside	6	2.3	0.0	0.0	0.0	0.6	1.6	0.0	4.5
	Outside	7	5.7	0.0	0.0	0.0	0.2	2.1	0.1	8.1
	Outside	8	4.3	0.0	0.0	0.0	0.2	0.6	0.1	5.2
Calatagan	Inside	1	7.5	0.0	0.0	0.2	0.2	0.3	0.3	8.5
	Inside	2	2.7	0.0	0.0	0.0	0.1	0.4	0.2	3.4
	Inside	3	5.0	0.0	0.0	0.0	0.6	0.4	0.4	6.3
	Inside	4	1.8	0.0	0.0	0.0	0.3	0.3	0.2	2.6
	Inside	5	3.3	0.0	0.0	0.0	0.7	0.0	0.3	4.2
	Outside	6	4.8	1.1	0.0	0.0	0.0	0.8	0.2	6.9
	Outside	7	2.0	0.0	0.1	0.0	0.1	0.9	0.2	3.3
	Outside	8	5.7	0.0	0.0	0.0	0.5	0.6	0.1	6.8
	Outside	9	3.4	0.0	0.0	0.0	0.4	0.2	0.0	4.0
	Outside	10	4.7	0.0	0.0	0.1	0.4	0.5	0.0	5.7
Mabini	Inside	1	13.8	0.0	0.0	2.6	1.1	9.6	12.3	39.4
	Inside	2	17.1	0.0	1.0	0.0	1.9	11.4	2.9	34.3
	Inside	3	18.2	0.0	0.0	2.0	1.1	5.1	5.0	31.3
	Inside	4	8.5	0.0	0.0	1.0	0.7	10.6	0.4	21.2
	Outside	5	5.3	0.0	0.0	0.1	0.5	4.5	0.6	11.2
	Outside	6	5.3	0.0	0.0	0.2	0.0	3.8	0.4	9.6
	Outside	7	5.6	0.0	0.3	3.8	0.6	0.8	0.0	11.1
	Outside	8	6.3	0.0	0.0	0.2	0.4	1.4	0.2	8.6
Tingloy	Inside	1	0.7	0.0	0.0	0.0	0.1	0.4	0.0	1.2
	Inside	2	13.5	0.0	0.0	7.1	0.0	6.3	2.1	29.0
	Inside	3	3.5	0.0	0.0	0.0	1.1	3.6	0.4	8.6
	Inside	4	5.8	0.0	0.0	0.2	0.3	1.0	0.4	7.7
	Outside	5	5.8	0.0	0.0	0.0	0.6	0.3	0.4	7.1
	Outside	6	6.8	3.7	0.0	0.6	1.8	0.9	1.3	15.2
	Outside	7	9.3	0.0	0.0	6.9	1.7	0.6	7.0	25.4
	Outside	8	2.0	0.0	0.0	0.0	0.7	2.4	0.3	5.4
Puerto Galera	Inside	1	0.5	0.0	3.7	0.0	0.5	5.8	0.0	10.5
	Inside	2	2.5	0.0	2.8	0.0	0.2	17.8	0.0	23.4
	Inside	3	2.8	0.0	1.6	0.0	0.7	6.1	0.2	11.4
	Outside	4	1.1	0.0	0.0	0.0	0.5	1.0	0.4	3.1
	Outside	5	2.5	0.0	0.0	0.0	0.8	1.1	0.0	4.5
	Outside	6	2.8	0.0	0.2	0.0	0.4	1.1	0.0	4.4
El Nido	Inside	1	3.9	0.0	0.0	0.3	0.1	8.3	0.5	13.1
	Inside	2	7.9	0.0	3.0	0.4	0.3	18.8	1.0	31.4
	Inside	3	2.2	0.0	1.5	0.6	0.2	13.7	3.6	21.9
	Inside	4	7.9	0.0	3.0	0.4	0.3	18.8	1.0	31.4
	Outside	5	2.6	0.0	1.1	3.0	0.9	4.8	0.9	13.3
	Outside	6	4.8	0.0	1.5	3.2	1.3	4.1	0.5	15.4
	Outside	7	7.6	0.0	0.0	1.6	0.9	11.5	0.9	22.5
	Outside	8	4.3	0.0	1.5	3.4	1.3	4.1	0.5	15.1
Dipaculao	Inside	1	2.4	0.5	0.0	0.1	0.0	1.5	0.5	5.0
	Inside	2	3.4	2.6	0.0	0.1	0.0	0.2	3.7	9.9
	Outside	3	4.3	1.6	0.0	1.6	0.2	5.9	0.1	13.7
	Outside	4	10.8	0.9	0.0	1.9	0.0	4.4	1.6	19.6
San Luis	Inside	1	1.9	0.0	0.0	0.0	0.0	0.3	0.0	2.2
	Inside	2	2.8	0.0	0.0	0.0	0.1	1.7	0.3	4.9
	Outside	3	2.6	0.0	0.0	0.0	0.0	0.0	0.6	3.2
	Outside	4	1.7	0.0	0.0	0.0	0.0	0.7	0.0	2.4
Siruma	Inside	1	0.3	0.0	0.0	5.0	0.0	6.8	2.2	14.4
	Inside	2	2.2	4.4	1.5	0.0	0.1	8.3	3.4	19.9
	Inside	3	6.6	0.0	0.0	0.0	0.9	5.8	14.1	27.4
	Inside	4	6.0	0.0	0.0	19.0	0.0	4.9	4.6	34.5
	Inside	5	1.2	0.7	0.0	0.0	2.1	9.4	7.8	21.3
	Outside	6	0.1	0.1	0.0	0.2	0.0	3.0	1.0	4.4
	Outside	7	0.5	0.0	0.0	0.9	0.0	2.3	0.1	3.8
	Outside	8	0.0	0.0	0.5	0.0	0.0	8.8	0.5	9.9
	Outside	9	2.0	0.0	0.0	13.2	0.0	5.1	0.5	20.9
	Outside	10	0.0	0.4	0.0	47.5	0.0	2.9	0.7	51.5
Tinambac	Inside	1	0.0	0.0	0.0	0.0	0.0	0.8	0.4	1.2
	Inside	2	1.6	0.0	0.0	0.0	0.0	15.9	1.7	19.2
	Inside	3	37.0	2.6	0.0	3.1	0.0	6.6	5.4	54.7
	Inside	4	6.7	0.0	0.0	0.4	0.0	9.2	0.3	16.5
	Inside	5	5.9	0.6	0.0	0.8	0.0	14.0	0.4	21.8
	Outside	6	0.0	0.0	0.0	0.0	0.0	2.0	1.3	3.4
	Outside	7	0.0	1.3	0.0	0.4	0.0	5.0	0.5	7.2
	Outside	8	0.0	0.0	0.0	0.0	0.0	8.8	0.0	8.8
	Outside	9	0.0	0.0	0.0	1.6	0.0	4.1	1.9	7.5
	Outside	10	0.0	0.0	0.0	0.2	0.0	7.6	0.2	8.0
Tigaon	Inside	1	6.8	1.6	0.0	32.0	0.0	0.2	0.5	41.2
	Inside	2	1.1	0.2	0.1	0.6	0.0	0.0	1.0	3.1
	Inside	3	0.5	1.2	0.2	0.3	0.5	0.1	0.4	3.3
	Outside	4	0.8	0.9	0.0	6.5	0.0	0.1	0.5	8.8
	Outside	5	0.4	0.4	0.0	7.3	0.1	0.4	0.0	8.6
	Outside	6	0.3	0.5	0.0	4.5	0.0	0.0	0.1	5.3
Sagnay	Inside	1	0.7	0.0	0.2	0.1	0.0	0.6	0.0	1.5
	Inside	2	0.7	0.2	0.0	0.1	0.0	0.4	0.0	1.5
	Inside	3	4.8	0.0	0.0	0.0	0.0	2.9	0.0	7.7
	Inside	4	0.8	0.0	0.0	0.0	0.0	0.3	0.0	1.1
	Inside	5	0.8	0.0	0.0	0.0	0.0	0.9	0.0	1.8
	Outside	6	1.3	0.0	0.0	0.0	0.0	0.5	0.1	1.9
	Outside	7	1.8	0.5	0.0	0.2	0.1	0.4	0.4	3.2
	Outside	8	60.0	0.0	0.1	0.0	0.3	0.7	0.1	61.2
	Outside	9	3.0	0.0	0.0	0.1	0.0	0.6	0.1	3.9
	Outside	10	3.2	0.0	0.0	0.0	0.0	0.6	0.0	3.9
Caramoan	Inside	1	2.4	0.0	0.0	1.9	0.0	2.8	0.9	7.9
	Inside	2	1.1	0.6	0.0	0.7	0.0	1.6	0.3	4.4
	Inside	3	1.4	1.4	0.0	1.5	0.0	6.4	3.7	14.3
	Inside	4	0.1	1.6	0.0	0.0	0.0	0.2	0.7	2.7
	Outside	5	0.0	0.5	0.0	0.4	0.0	2.1	0.1	3.2
	Outside	6	0.0	0.0	0.0	0.0	0.0	4.0	0.1	4.1
	Outside	7	0.0	0.0	0.0	0.0	0.0	0.7	0.1	0.9
	Outside	8	0.3	0.0	0.0	0.0	0.0	0.9	0.2	1.4
Bacacay	Inside	1	2.5	1.5	0.0	0.0	0.0	2.2	0.1	6.3
	Inside	2	2.9	0.0	0.0	0.0	0.1	0.4	0.0	3.5
	Inside	3	2.4	0.0	0.0	0.0	0.0	0.2	0.0	2.7
	Inside	4	2.3	0.0	0.0	0.0	0.0	0.0	0.0	2.3
	Outside	5	1.3	0.0	0.0	0.0	0.1	0.7	0.0	2.1
	Outside	6	3.2	0.0	0.0	0.0	0.0	0.9	0.0	4.0
	Outside	7	0.8	0.0	0.0	0.0	0.0	0.1	0.0	0.9
	Outside	8	1.6	0.0	0.0	0.0	0.1	1.2	0.0	2.9
Gubat	Inside	1	2.2	0.0	0.0	0.0	0.0	0.2	0.0	2.4
	Inside	2	2.7	0.0	0.0	0.0	0.0	3.7	0.0	6.4
	Inside	3	5.9	0.0	0.0	0.0	0.1	6.1	0.4	12.5
	Inside	4	6.8	0.0	0.0	0.0	0.0	4.8	0.0	11.6
	Inside	5	0.7	0.6	0.0	0.0	0.0	1.5	0.0	2.7
	Outside	6	0.8	0.0	0.0	0.3	0.0	0.6	0.0	1.6
	Outside	7	1.5	0.3	0.0	0.0	0.3	2.6	0.0	4.7
	Outside	8	2.9	0.0	0.0	0.0	0.0	3.7	0.0	6.6
	Outside	9	5.2	0.0	0.0	0.0	0.2	3.3	0.0	8.7
	Outside	10	12.2	0.0	0.0	0.1	0.0	0.8	0.0	13.1
Matnog	Inside	1	1.6	0.0	0.0	0.2	0.0	0.7	0.0	2.6
	Inside	2	0.2	0.0	0.0	0.0	0.0	0.3	0.0	0.5
	Inside	3	0.5	0.0	0.0	0.0	0.0	0.0	0.0	0.5
	Inside	4	0.1	0.0	0.0	0.0	0.1	0.0	0.0	0.2
	Outside	5	0.5	0.0	0.0	0.0	0.1	0.0	0.0	0.7
	Outside	6	17.3	0.0	0.2	0.0	0.0	1.1	0.0	18.7
	Outside	7	0.5	0.0	0.0	0.0	0.0	3.0	0.0	3.5
	Outside	8	1.5	0.0	0.0	0.0	0.0	2.3	0.0	3.7
Hinunangan	Inside	1	3.2	0.2	0.0	0.0	0.0	9.8	0.3	13.5
	Inside	2	3.8	0.1	0.0	0.0	0.0	3.1	0.0	7.0
	Inside	3	0.5	0.0	0.0	0.0	0.0	0.1	0.2	0.7
	Inside	4	0.8	0.0	0.0	0.0	0.0	2.7	0.1	3.6
	Inside	5	0.2	0.0	0.0	0.1	0.0	0.7	0.3	1.4
	Outside	6	0.8	0.0	0.0	0.0	0.0	0.1	0.2	1.1
	Outside	7	1.6	0.0	0.0	0.0	0.0	1.0	0.2	2.8
	Outside	8	0.0	0.0	0.0	0.0	0.0	0.0	0.1	0.2
	Outside	9	0.1	0.0	0.0	0.0	0.0	1.0	0.5	1.6
	Outside	10	0.4	0.0	0.0	0.0	0.0	0.3	0.0	0.7
San Francisco, Leyte	Inside	1	1.9	0.0	0.0	1.8	1.2	6.6	3.4	15.0
	Inside	2	0.6	0.0	0.0	0.7	0.0	3.9	2.5	7.7
	Inside	3	0.0	0.0	0.0	0.0	0.0	2.0	0.9	2.9
	Inside	4	0.0	0.0	0.0	0.0	1.8	0.1	0.3	2.2
	Inside	5	0.0	0.0	0.0	0.0	0.0	0.5	0.0	0.5
	Outside	6	0.2	0.0	0.0	0.9	0.0	2.5	0.2	3.8
	Outside	7	0.0	0.0	0.0	0.2	0.0	0.3	0.1	0.5
	Outside	8	0.0	0.0	0.0	1.7	0.0	0.9	0.1	2.7
	Outside	9	0.0	0.0	0.0	0.0	0.0	0.3	0.0	0.3
	Outside	10	0.0	0.0	0.0	0.0	0.0	0.9	0.0	0.9
Cantilan	Inside	1	2.8	0.0	0.2	0.7	0.4	1.8	0.0	6.0
	Inside	2	9.2	0.0	0.0	0.0	0.4	5.8	0.0	15.3
	Inside	3	2.9	0.0	0.0	0.1	1.4	0.8	0.8	6.0
	Inside	4	5.7	0.0	0.3	0.4	0.0	5.1	0.0	11.5
	Outside	5	1.2	0.0	0.0	0.3	0.0	0.1	1.1	2.6
	Outside	6	1.1	0.1	1.4	0.0	0.0	1.8	0.0	4.4
	Outside	7	2.5	0.0	0.0	0.0	0.3	0.6	0.4	3.8
	Outside	8	1.6	0.0	0.0	0.2	0.3	0.9	0.0	3.0
Lanuza	Inside	1	0.8	0.0	0.0	0.0	0.0	4.0	0.0	4.9
	Inside	2	2.4	0.0	0.2	0.0	0.1	2.4	0.0	5.1
	Inside	3	0.6	0.0	0.4	0.0	0.5	0.0	0.0	1.5
	Inside	4	2.0	0.0	1.6	0.0	0.0	0.6	0.0	4.2
	Inside	5	0.9	0.0	0.0	0.5	0.0	1.3	0.0	2.7
	Outside	6	0.6	0.0	0.0	0.0	0.0	0.9	0.0	1.5
	Outside	7	0.3	0.0	0.0	0.0	0.0	3.6	0.0	3.9
	Outside	8	3.3	0.0	0.0	0.0	0.0	3.8	0.0	7.1
	Outside	9	2.7	0.0	0.0	0.3	0.0	1.8	0.0	4.8
	Outside	10	2.5	0.0	0.0	0.0	0.0	0.3	0.0	2.8
Cortes	Inside	1	4.1	0.0	0.0	0.0	0.4	0.4	0.0	4.9
	Inside	2	13.0	0.0	0.0	1.1	0.9	4.3	0.2	19.5
	Inside	3	1.3	0.0	0.3	0.0	0.1	1.3	0.0	2.9
	Inside	4	8.2	0.0	0.0	0.0	0.3	0.1	0.0	8.5
	Inside	5	1.5	0.0	0.0	0.0	0.9	0.7	0.0	3.2
	Outside	6	0.3	0.0	0.0	0.0	0.1	0.3	0.0	0.7
	Outside	7	0.3	0.0	0.0	0.0	0.0	0.7	0.0	1.0
	Outside	8	5.1	0.0	0.0	0.0	0.2	0.2	0.0	5.4
	Outside	9	1.1	0.0	0.0	17.0	0.1	0.5	0.0	18.7
	Outside	10	2.3	0.0	0.6	0.4	0.2	0.2	0.0	3.7
Lianga	Inside	1	0.4	0.0	0.0	0.1	0.0	1.7	0.0	2.2
	Inside	2	0.2	0.0	0.0	0.0	0.0	0.7	0.0	0.9
	Inside	3	2.0	0.0	0.3	1.3	0.0	4.4	0.0	8.1
	Inside	4	0.0	0.0	0.0	0.0	0.1	0.6	0.0	0.8
	Inside	5	0.2	0.0	0.0	0.0	0.3	0.4	0.0	0.8
	Outside	6	0.3	0.0	0.0	0.0	0.1	0.2	0.0	0.6
	Outside	7	0.3	0.0	0.0	0.0	0.0	0.0	0.0	0.3
	Outside	8	0.0	0.0	0.0	0.0	0.0	0.3	0.0	0.3
	Outside	9	0.4	0.0	0.0	0.0	0.0	0.3	0.0	0.7
	Outside	10	2.5	0.0	1.6	0.3	0.0	4.6	0.0	9.1
Marihatag	Inside	1	0.2	1.1	0.0	0.0	0.0	3.6	0.3	5.1
	Inside	2	2.9	0.3	0.0	0.6	1.2	7.7	0.1	12.8
	Inside	3	0.1	0.0	0.0	0.0	0.0	0.0	0.0	0.1
	Inside	4	0.3	0.0	0.0	0.0	0.0	0.0	0.0	0.3
	Outside	5	0.0	0.0	0.0	0.0	0.0	0.0	0.0	0.0
	Outside	6	0.1	0.0	0.0	0.0	0.2	0.3	0.0	0.6
	Outside	7	0.1	0.0	0.0	0.0	0.1	0.0	0.0	0.2
	Outside	8	0.5	0.0	0.0	0.2	0.1	1.3	0.0	2.0
Mati City	Inside	1	0.6	0.0	0.0	0.0	0.0	0.1	0.3	1.0
	Inside	2	33.6	0.0	0.0	0.0	0.5	0.0	0.2	34.3
	Inside	3	0.7	0.0	0.0	0.0	0.3	0.6	0.0	1.7
	Inside	4	6.8	0.0	0.9	0.0	0.8	0.5	0.2	9.2
	Inside	5	0.6	0.0	0.0	0.0	0.0	1.5	0.0	2.2
	Outside	6	1.2	0.0	0.1	0.0	0.1	0.4	0.0	1.8
	Outside	7	1.7	0.0	0.1	0.0	0.0	0.0	0.1	1.9
	Outside	8	5.5	0.0	0.0	0.1	0.0	1.0	0.1	6.6
	Outside	9	1.2	0.0	0.3	0.0	0.0	0.1	0.0	1.7
	Outside	10	3.9	0.0	0.2	0.0	0.1	1.3	0.4	5.8
Roxas	Inside	1	3.2	0.0	0.0	1.6	0.4	1.0	0.2	6.4
	Inside	2	0.8	0.4	0.3	0.6	0.8	0.9	0.5	4.4
	Inside	3	0.3	0.4	0.0	0.5	0.0	0.9	0.1	2.1
	Inside	4	0.3	0.0	0.0	0.8	0.2	0.6	0.6	2.6
	Outside	5	2.4	0.0	0.0	1.3	0.3	0.0	1.3	5.4
	Outside	6	1.0	1.3	0.7	1.1	2.2	9.8	0.4	16.5
	Outside	7	1.8	0.8	0.0	0.7	0.6	0.3	0.6	4.6
	Outside	8	1.0	0.9	0.6	1.1	0.4	1.9	0.2	6.1
Bulalacao	Inside	1	0.1	0.0	0.0	0.0	0.0	4.7	0.0	4.8
	Inside	2	0.2	0.0	0.0	0.0	0.0	2.2	0.1	2.5
	Inside	3	0.4	0.0	0.0	0.0	0.1	1.1	0.1	1.7
	Outside	4	0.1	0.3	0.0	0.0	0.1	1.3	0.1	1.9
	Outside	5	0.0	0.0	0.0	0.0	0.2	1.1	0.0	1.3
	Outside	6	0.1	0.0	0.0	0.0	0.2	4.9	0.0	5.3
Siaton	Inside	1	0.9	0.0	0.0	0.0	0.6	0.0	4.7	6.2
	Inside	2	14.2	0.0	0.6	1.3	4.4	2.1	3.0	25.4
	Inside	3	2.6	0.0	0.0	0.0	2.1	0.6	2.4	7.6
	Inside	4	24.4	0.0	1.1	0.5	2.7	0.0	0.7	29.4
	Outside	5	1.6	0.0	0.0	0.0	0.5	0.2	0.7	3.0
	Outside	6	3.8	0.0	0.0	0.0	1.3	2.7	0.3	8.2
	Outside	7	4.0	0.0	0.0	0.4	1.9	0.8	3.5	10.6
	Outside	8	5.2	0.0	0.0	0.2	2.4	1.5	0.3	9.5
Bongao	Inside	1	12.4	0.0	0.0	0.0	0.0	10.8	1.0	24.2
	Inside	2	6.5	0.9	0.0	0.0	0.4	0.8	1.9	10.6
	Inside	3	12.5	0.1	0.0	0.0	0.6	0.6	0.1	13.9
	Outside	4	11.8	0.0	0.8	1.9	0.5	2.3	0.2	17.4
	Outside	5	5.9	22.0	6.5	5.9	1.1	2.4	2.9	46.7
	Outside	6	0.0	0.0	0.8	0.0	0.0	0.2	0.2	1.2
Panglima Sugala	Inside	1	11.8	31.0	0.0	0.0	0.0	19.0	1.3	63.2
	Inside	2	2.3	1.3	0.0	0.5	0.5	4.8	0.5	9.8
	Inside	3	4.7	0.0	0.4	10.9	1.8	3.1	0.0	20.9
	Outside	4	3.2	1.7	0.4	0.7	0.8	6.9	0.7	14.4
	Outside	5	5.2	1.1	0.0	0.0	0.0	2.2	0.0	8.4
	Outside	6	2.4	2.0	0.0	0.0	0.6	1.1	0.3	6.4
Simunul	Inside	1	5.2	0.3	0.0	0.0	0.3	2.6	0.1	8.6
	Inside	2	42.5	2.6	2.4	1.3	1.6	10.4	2.6	63.3
	Inside	3	3.4	0.0	0.0	0.0	0.1	0.8	0.4	4.7
	Outside	4	9.9	0.6	1.0	0.0	0.2	2.9	1.2	15.9
	Outside	5	6.6	0.3	0.8	0.4	0.4	1.5	0.1	10.0
	Outside	6	35.2	0.0	0.0	0.3	0.1	6.0	1.0	42.6
Calapan City	Inside	1	5.5	0.0	0.0	0.0	0.0	1.9	0.0	7.4
	Inside	2	5.7	0.0	0.0	0.0	0.1	2.2	0.0	8.1
	Inside	3	13.6	0.0	0.0	0.0	0.2	2.8	0.1	16.7
	Outside	4	0.0	0.0	0.0	0.0	0.7	0.0	0.0	0.7
	Outside	5	1.0	0.0	0.0	0.0	0.7	0.0	0.0	1.6
	Outside	6	5.9	0.0	0.0	0.0	0.6	0.0	0.0	6.6
Naujan	Inside	1	1.3	0.0	0.0	0.0	0.3	8.5	0.0	10.0
	Inside	2	1.4	0.0	0.0	0.0	0.3	5.7	0.1	7.4
	Inside	3	5.3	0.0	0.0	0.0	0.4	15.3	0.1	21.1
	Outside	4	0.3	0.0	0.0	0.1	0.1	0.2	0.0	0.7
	Outside	5	0.2	0.0	0.0	0.2	0.0	0.0	0.0	0.4
	Outside	6	1.8	0.0	0.0	0.0	0.1	0.0	0.0	1.9
Pola	Inside	1	4.2	5.3	0.0	0.2	0.6	5.2	0.0	15.5
	Inside	2	3.1	0.0	0.3	0.4	0.4	4.9	0.4	9.6
	Inside	3	2.6	0.0	0.0	1.7	1.6	2.7	0.1	8.8
	Outside	4	2.3	0.0	0.0	0.0	0.3	0.2	0.0	2.7
	Outside	5	5.4	0.0	0.0	0.2	0.0	4.3	0.0	9.9
	Outside	6	5.2	0.0	0.0	0.6	0.1	0.0	0.0	5.9
Gloria	Inside	1	13.9	44.5	0.0	1.4	0.0	2.4	0.0	62.3
	Inside	2	8.8	0.0	0.6	4.1	0.9	14.5	0.9	29.7
	Inside	3	7.3	6.2	0.0	12.9	4.1	4.8	1.5	36.9
	Outside	4	1.1	0.8	0.0	0.1	0.4	0.0	0.0	2.4
	Outside	5	2.1	0.4	0.0	0.0	0.1	0.1	0.8	3.5
	Outside	6	1.5	0.4	0.0	0.2	0.2	0.7	0.0	3.0
Bongabong	Inside	1	0.3	0.0	0.0	0.0	0.0	0.0	0.0	0.3
	Inside	2	4.3	0.0	0.0	0.0	0.0	0.0	0.0	4.3
	Inside	3	0.8	0.0	0.0	0.3	0.0	0.1	0.0	1.1
	Outside	4	0.0	0.0	0.0	0.0	0.0	0.0	0.1	0.1
	Outside	5	0.1	0.0	0.0	0.0	0.0	0.0	0.0	0.1
	Outside	6	0.4	0.0	0.0	0.1	0.0	0.0	0.0	0.6
Mansalay	Inside	1	0.7	0.0	0.0	0.0	0.2	5.3	0.0	6.2
	Inside	2	0.5	0.0	0.0	0.0	0.0	0.2	0.1	0.8
	Inside	3	0.3	0.0	0.0	0.1	0.0	0.3	0.1	0.8
	Outside	4	0.1	0.0	0.0	0.0	0.1	0.5	0.0	0.8
	Outside	5	1.6	0.0	0.0	0.0	0.3	0.0	0.0	1.9
	Outside	6	0.1	0.0	0.0	0.0	0.1	0.0	0.0	0.2
Bulan	Inside	1	0.0	0.2	0.0	0.0	0.4	5.1	0.2	5.9
	Inside	2	0.1	0.2	0.0	0.0	0.0	3.3	0.1	3.7
	Inside	3	0.0	0.0	0.0	0.0	0.0	0.0	0.3	0.3
	Inside	4	0.0	0.1	0.0	0.0	0.0	0.1	0.1	0.3
	Inside	5	0.0	0.0	0.1	0.0	0.0	0.1	0.1	0.2
	Outside	6	0.0	0.2	0.0	0.0	0.3	0.3	0.1	0.9
	Outside	7	0.0	0.0	0.0	0.0	0.0	0.0	0.0	0.1
	Outside	8	0.0	0.0	0.0	0.0	0.3	0.6	0.2	1.0
	Outside	9	0.0	0.0	0.0	0.0	0.2	0.2	0.1	0.5
	Outside	10	0.0	0.2	0.0	0.0	0.0	0.3	0.3	0.8
Ayungon	Inside	1	2.8	0.0	0.0	0.1	0.7	1.7	0.5	5.9
	Inside	2	19.4	0.0	0.0	4.4	1.2	14.9	0.3	40.2
	Inside	3	5.9	0.0	0.0	0.8	0.0	4.6	1.3	12.7
	Inside	4	9.2	0.0	0.0	0.3	0.3	13.6	0.5	23.8
	Inside	5	3.0	0.0	0.0	0.6	0.2	5.2	0.3	9.4
	Outside	6	0.0	0.0	0.0	0.4	0.1	0.1	0.5	1.1
	Outside	7	0.0	0.0	0.0	0.6	0.2	0.0	0.6	1.4
	Outside	8	0.8	0.0	0.0	0.0	0.2	0.4	0.5	1.9
	Outside	9	6.3	0.0	0.0	0.0	0.0	1.4	0.7	8.4
	Outside	10	2.9	0.0	0.0	0.0	0.0	0.0	0.2	3.1
Bindoy	Inside	1	4.7	0.0	0.0	0.3	0.2	3.6	0.4	9.2
	Inside	2	2.8	0.0	0.0	0.1	0.1	3.7	0.4	7.1
	Inside	3	3.3	0.0	0.0	0.0	0.5	4.6	1.2	9.6
	Inside	4	1.6	0.0	0.0	0.2	0.0	4.2	1.3	7.3
	Inside	5	11.3	0.0	0.0	0.3	0.3	5.1	0.2	17.2
	Outside	6	4.1	0.0	0.0	0.1	0.0	0.4	0.0	4.5
	Outside	7	12.0	0.0	0.0	0.0	0.8	2.7	0.1	15.6
	Outside	8	3.6	0.0	0.0	0.0	0.0	0.6	0.2	4.4
	Outside	9	3.6	0.0	0.0	0.0	0.0	1.0	0.1	4.8
	Outside	10	14.2	0.0	0.0	0.0	0.0	0.8	0.8	15.8
Amlan	Inside	1	4.7	0.0	0.0	4.6	1.1	10.2	6.4	27.0
	Inside	2	14.1	11.5	0.0	50.3	1.5	23.8	3.1	104.2
	Inside	3	1.7	0.0	0.0	0.5	0.3	2.3	0.1	4.7
	Inside	4	1.2	0.0	0.0	1.6	0.2	0.5	0.0	3.6
	Inside	5	0.8	0.0	0.0	0.0	0.5	1.3	0.0	2.6
	Outside	6	0.0	0.0	0.0	0.0	1.6	0.4	0.0	2.0
	Outside	7	0.8	0.0	0.0	0.0	0.0	2.0	0.1	2.9
	Outside	8	0.8	0.0	0.0	0.0	0.2	0.2	0.0	1.2
	Outside	9	1.4	0.0	0.0	0.0	0.1	0.0	0.0	1.5
	Outside	10	2.8	0.0	0.0	0.2	0.2	0.0	0.0	3.2
San Francisco, Cebu	Inside	1	5.3	0.0	0.0	1.2	1.3	2.2	0.2	10.2
	Inside	2	20.8	0.0	0.0	0.0	1.3	1.3	0.0	23.3
	Inside	3	6.4	2.7	0.0	5.2	2.4	3.4	0.1	20.2
	Inside	4	26.6	0.0	0.0	9.8	5.5	12.9	0.2	54.9
	Inside	5	4.3	0.0	0.0	0.5	2.1	5.4	0.4	12.6
	Outside	6	0.5	0.0	0.0	0.0	0.7	0.0	0.0	1.3
	Outside	7	11.7	0.0	0.6	0.4	1.6	1.2	0.1	15.5
	Outside	8	2.1	0.0	0.0	0.0	0.4	0.2	0.2	2.8
	Outside	9	1.9	0.1	0.0	0.0	0.6	0.1	0.1	2.8
	Outside	10	3.3	0.0	0.0	0.0	0.8	1.8	0.2	6.1
Pilar	Inside	1	2.0	0.0	0.0	0.0	0.0	0.0	0.2	2.2
	Inside	2	3.2	0.0	0.0	0.1	0.1	0.8	0.3	4.6
	Inside	3	0.0	0.0	0.0	0.0	0.2	0.1	0.0	0.4
	Inside	4	3.4	0.0	0.0	0.7	0.3	0.7	0.1	5.1
	Inside	5	13.1	0.0	0.0	2.3	0.2	1.3	0.2	17.0
	Outside	6	0.3	0.0	0.0	0.0	0.1	0.0	0.0	0.4
	Outside	7	0.4	0.0	0.0	0.6	0.1	0.0	0.0	1.1
	Outside	8	0.4	0.0	0.0	0.0	0.0	0.4	0.4	1.2
	Outside	9	2.4	0.0	0.0	0.0	0.2	1.6	0.4	4.6
	Outside	10	2.8	0.0	0.0	0.3	0.5	0.9	0.2	4.6
Boljoon	Inside	1	2.0	0.0	0.8	0.0	0.4	5.9	1.4	10.4
	Inside	2	4.0	0.0	0.8	2.5	1.0	8.1	1.0	17.3
	Inside	3	1.0	0.0	0.0	0.8	0.0	3.8	0.6	6.2
	Inside	4	4.2	0.0	0.0	0.0	0.0	7.9	0.3	12.5
	Inside	5	2.3	0.0	0.3	1.1	0.1	5.0	0.1	8.9
	Outside	6	0.8	0.0	0.0	0.0	4.1	8.7	0.0	13.7
	Outside	7	1.2	0.0	0.0	0.3	0.0	0.9	0.2	2.6
	Outside	8	2.9	0.0	0.0	0.3	0.2	13.3	0.2	17.0
	Outside	9	2.0	0.0	0.0	0.2	0.3	3.6	0.4	6.5
	Outside	10	14.1	0.0	0.0	0.4	0.5	8.0	0.5	23.5
Ubay	Inside	1	0.0	0.0	0.0	0.2	0.0	0.4	0.2	0.8
	Inside	2	0.0	0.1	0.0	2.4	0.1	0.0	0.7	3.3
	Inside	3	0.6	0.0	0.0	0.0	0.2	0.1	0.1	1.0
	Inside	4	0.0	0.0	0.0	0.0	0.0	0.0	0.2	0.3
	Inside	5	0.0	0.1	0.0	0.6	0.2	4.4	0.2	5.5
	Outside	6	0.1	0.0	0.0	0.0	0.1	0.6	0.0	0.8
	Outside	7	0.0	0.0	0.1	0.0	0.0	0.1	0.0	0.2
	Outside	8	0.0	0.0	0.0	0.0	0.0	0.0	0.1	0.1
	Outside	9	0.0	0.0	0.0	0.0	0.2	1.1	0.2	1.5
	Outside	10	0.0	0.0	0.0	0.0	0.4	0.8	0.4	1.7
Inabanga	Inside	1	0.0	1.2	0.0	0.0	0.1	3.9	0.9	6.1
	Inside	2	0.1	0.0	0.0	0.3	0.6	28.5	1.1	30.6
	Inside	3	0.0	0.0	0.0	1.4	0.2	5.2	6.8	13.6
	Inside	4	0.0	0.0	0.0	0.6	0.0	10.0	2.5	13.1
	Inside	5	0.4	0.0	0.0	0.0	0.0	4.7	0.9	6.0
	Outside	6	0.0	0.0	0.0	0.0	0.0	0.2	0.1	0.2
	Outside	7	0.0	0.0	0.0	0.0	0.0	0.3	0.0	0.3
	Outside	8	0.0	0.1	0.0	0.0	0.2	0.0	0.1	0.4
	Outside	9	0.0	0.0	0.0	0.0	0.0	0.1	0.0	0.1
	Outside	10	0.0	2.1	0.0	0.0	0.7	0.0	0.1	3.0
Tagbilaran City	Inside	1	3.7	0.0	1.2	0.6	0.4	4.1	0.4	10.4
	Inside	2	19.0	0.0	0.0	1.1	0.0	3.8	0.2	24.2
	Inside	3	0.1	0.0	0.0	0.0	0.6	4.5	0.2	5.4
	Inside	4	0.3	0.0	0.0	0.0	0.4	3.1	1.0	4.8
	Inside	5	1.4	0.0	0.0	7.2	1.1	5.3	0.7	15.8
	Outside	6	0.0	0.0	0.0	0.0	0.1	1.8	0.4	2.3
	Outside	7	0.0	0.0	0.0	0.0	0.1	0.5	1.2	1.8
	Outside	8	0.1	0.0	0.0	0.0	0.0	2.3	1.0	3.4
	Outside	9	0.0	0.0	0.0	0.0	0.1	0.5	1.2	1.8
	Outside	10	0.0	0.0	0.0	0.1	0.0	1.4	2.2	3.7
Panabo City	Inside	1	0.0	0.0	0.0	0.0	0.0	0.0	0.0	0.0
	Inside	2	0.0	0.0	0.0	0.0	0.0	0.0	0.0	0.0
	Inside	3	0.0	0.0	0.0	0.0	0.0	0.9	0.0	0.9
	Inside	4	0.2	0.0	0.0	0.0	0.0	0.0	0.0	0.2
	Outside	5	0.0	0.0	0.0	0.0	0.0	0.0	0.0	0.0
	Outside	6	0.0	0.0	0.0	0.0	0.0	0.0	0.0	0.0
	Outside	7	0.0	0.0	0.0	0.0	0.0	0.1	0.0	0.1
	Outside	8	0.2	0.0	0.0	0.0	0.0	0.3	0.0	0.6
Dapia, IGACOS	Inside	1	1.6	0.0	0.0	0.0	0.0	2.9	0.0	4.5
	Inside	2	17.9	0.0	0.0	0.0	0.0	1.8	0.0	19.7
	Inside	3	0.3	0.0	0.0	0.0	0.0	0.1	0.0	0.4
	Inside	4	1.2	0.0	0.0	0.0	0.0	0.5	0.0	1.7
	Inside	5	18.0	0.0	0.0	0.0	0.0	0.9	0.1	19.0
	Outside	6	6.2	0.0	0.0	0.0	0.0	0.6	0.0	6.8
	Outside	7	17.3	0.0	0.0	0.5	0.0	2.4	0.0	20.1
	Outside	8	0.4	0.0	0.0	0.0	0.0	1.7	0.0	2.1
	Outside	9	6.3	0.0	0.0	0.0	0.0	1.4	0.1	7.8
	Outside	10	0.7	0.0	0.0	0.0	0.2	0.0	0.1	1.0
Sanipaan, IGACOS	Inside	1	0.7	0.0	0.0	0.0	0.0	0.5	0.1	1.4
	Inside	2	48.1	0.0	0.0	0.0	0.0	0.7	0.2	49.0
	Inside	3	2.5	0.0	0.0	0.0	0.0	1.4	0.0	3.8
	Inside	4	0.3	0.0	0.0	0.0	0.0	0.7	0.0	1.0
	Inside	5	0.3	2.4	0.0	0.0	0.0	0.4	0.0	3.1
	Outside	6	0.7	0.0	0.0	0.0	0.0	0.1	0.0	0.8
	Outside	7	0.3	0.0	0.0	0.0	0.0	0.1	0.2	0.5
	Outside	8	5.1	0.6	0.0	0.0	0.2	0.1	0.0	5.9
	Outside	9	19.5	0.0	0.0	0.0	0.0	0.9	0.0	20.4
	Outside	10	0.0	0.0	0.0	0.0	0.0	0.0	0.0	0.0
Dumalinao	Inside	1	0.6	0.9	0.0	0.0	1.4	2.2	0.4	5.6
	Inside	2	1.7	0.0	0.0	0.0	1.2	7.8	0.0	10.7
	Outside	3	1.9	2.5	0.0	0.3	0.2	0.3	0.2	5.5
	Outside	4	0.0	0.0	0.0	0.0	0.0	0.0	0.0	0.0
Tabina	Inside	1	10.7	2.6	0.0	1.6	3.0	12.0	1.1	31.0
	Inside	2	5.8	0.0	2.5	2.4	1.8	9.4	1.6	23.6
	Inside	3	8.3	0.0	1.3	3.1	0.9	6.1	5.8	25.5
	Inside	4	3.3	0.0	5.2	1.5	0.7	2.1	1.5	14.3
	Outside	5	2.9	0.0	0.0	0.7	0.3	8.0	0.0	11.9
	Outside	6	2.9	0.0	0.0	0.7	0.3	8.0	0.0	11.9
	Outside	7	13.6	0.0	0.0	0.0	0.5	4.7	0.2	18.9
	Outside	8	3.6	0.0	0.0	0.0	0.4	6.9	0.0	10.9
Tukuran	Inside	1	0.9	0.0	0.0	0.0	0.7	2.1	0.1	3.8
	Inside	2	1.7	0.0	0.7	0.0	0.8	3.9	0.1	7.2
	Inside	3	0.9	0.0	0.0	13.1	0.6	0.7	0.1	15.4
	Inside	4	0.9	0.0	0.0	10.3	0.6	0.5	0.1	12.3
	Outside	5	0.5	0.0	0.0	0.0	0.0	1.8	0.0	2.4
	Outside	6	0.4	0.0	0.0	0.0	0.0	0.2	0.0	0.6
	Outside	7	0.6	0.0	0.0	0.4	0.0	0.0	0.5	1.4
	Outside	8	1.4	0.0	0.0	0.0	0.1	0.0	0.1	1.6
Ipil	Inside	1	2.8	1.6	0.0	5.5	0.0	0.0	0.1	10.0
	Inside	2	1.1	0.0	0.0	0.5	0.0	0.8	1.2	3.6
	Inside	3	0.0	0.0	0.0	0.0	0.0	4.7	1.6	6.3
	Inside	4	0.0	0.1	0.0	2.1	0.0	1.4	0.3	3.8
	Inside	5	0.2	0.0	0.0	1.8	0.0	1.4	0.3	3.8
	Outside	6	0.0	0.0	0.0	1.2	1.2	0.6	0.1	3.0
	Outside	7	0.0	0.0	0.0	2.2	0.0	1.1	0.4	3.7
	Outside	8	0.0	0.0	0.0	5.9	0.1	2.2	0.3	8.5
	Outside	9	0.8	0.0	0.0	9.3	0.0	1.2	0.0	11.4
	Outside	10	0.0	0.0	0.0	0.3	0.2	0.0	0.1	0.6
TRNMP, Cagayancillo	Inside	1	165.0	0.0	1.9	0.9	0.1	0.3	10.5	178.7
	Inside	2	33.2	0.0	5.3	0.6	1.3	4.6	2.8	47.7
	Inside	3	24.6	0.0	1.7	2.9	2.6	0.2	5.3	37.3
	Inside	4	28.7	0.0	0.4	4.9	0.8	6.6	18.0	59.5
	Inside	5	2.9	0.0	1.1	0.0	1.1	0.0	7.6	12.7
	Inside	6	6.6	0.0	1.4	0.1	0.2	5.3	6.0	19.7
	Inside	7	16.6	0.0	0.4	0.7	1.3	2.6	6.1	27.8
	Inside	8	7.6	0.0	0.3	1.8	0.3	0.5	2.3	12.8
	Inside	9	44.4	0.0	4.5	4.1	0.1	4.6	9.4	67.0
	Inside	10	53.3	0.0	2.3	0.9	0.2	1.8	6.4	65.0
	Inside	11	26.4	0.0	0.4	0.5	0.0	7.4	2.8	37.4
	Inside	12	15.9	0.0	1.3	3.8	1.7	6.1	16.9	45.7
	Inside	13	8.2	0.0	4.6	4.7	1.1	8.5	5.0	32.1
	Inside	14	7.0	0.0	0.0	0.0	0.0	5.5	3.5	16.0
	Inside	15	7.9	0.0	0.0	0.0	0.6	7.3	6.0	21.8
	Inside	16	15.9	0.0	0.0	0.0	0.9	4.5	3.3	24.5
	Inside	17	18.8	5.2	32.5	31.2	2.0	8.1	2.2	100.0
	Inside	18	20.9	0.0	0.3	64.0	4.1	12.2	3.0	104.6
	Inside	19	73.6	73.4	0.0	7.7	0.0	8.0	3.9	166.5
	Inside	20	43.8	1.7	0.5	9.7	0.0	11.2	4.5	71.4
	Inside	21	21.2	0.0	10.4	3.5	0.1	6.6	1.7	43.5
	Inside	22	34.3	0.0	5.9	3.2	0.7	6.1	3.1	53.4
	Inside	23	10.5	7.4	0.5	2.7	0.3	4.7	3.2	29.4
	Inside	24	5.7	0.0	0.8	8.8	0.4	1.8	4.9	22.5
	Inside	25	23.6	0.0	0.8	2.2	0.2	11.9	3.5	42.2
	Inside	26	16.7	0.0	2.7	2.5	0.1	3.8	1.7	27.6
	Inside	27	3.6	0.0	0.0	0.0	0.5	2.7	3.8	10.6
	Inside	28	12.4	0.0	16.4	0.3	0.6	5.7	4.8	40.2
	Inside	29	37.0	1.7	0.0	1.1	0.0	6.6	7.3	53.8
	Inside	30	55.2	0.0	21.4	20.5	0.0	19.1	6.0	122.2
	Inside	31	20.9	0.9	4.5	11.4	1.0	12.7	5.8	57.2
	Inside	32	13.1	0.0	4.8	0.3	0.4	10.8	5.0	34.5
	Inside	33	8.9	0.0	10.2	83.3	2.4	13.7	14.2	132.6

**Table 2:** Mean biomass (mt/km^2^) of commercially important coral reef fishes inside and outside MPAs.

Municipality	MPA zone	Acanthuridae	Haemulidae	Lethrinidae	Lutjanidae	Mullidae	Scarinae	Epinephelinae	Total
San Fernando City	Inside	1.2	0.0	0.0	0.2	0.2	0.2	1.1	2.9
	Outside	1.3	0.0	0.2	0.0	0.1	0.6	0.3	2.4
Alaminos City	Inside	0.5	0.0	0.0	0.2	0.2	1.1	0.2	2.3
	Outside	3.2	0.3	0.0	0.3	0.3	1.8	0.1	6.0
Bolinao	Inside	4.1	0.2	0.1	0.7	0.2	1.0	0.6	6.9
	Outside	3.9	0.3	0.0	0.2	0.6	0.7	0.6	6.3
Candelaria	Inside	3.3	0.0	0.0	0.0	0.6	2.2	0.9	6.9
	Outside	0.6	0.0	0.0	0.0	0.1	0.6	0.2	1.4
Masinloc	Inside	10.0	0.0	0.4	0.6	0.7	2.5	0.2	14.3
	Outside	3.5	0.0	0.1	0.0	0.3	1.2	0.1	5.3
Calatagan	Inside	4.1	0.0	0.0	0.0	0.4	0.3	0.3	5.0
	Outside	4.1	0.2	0.0	0.0	0.3	0.6	0.1	5.3
Mabini	Inside	14.4	0.0	0.2	1.4	1.2	9.2	5.1	31.6
	Outside	5.6	0.0	0.1	1.1	0.4	2.7	0.3	10.1
Tingloy	Inside	5.8	0.0	0.0	1.8	0.4	2.8	0.7	11.6
	Outside	6.0	0.9	0.0	1.9	1.2	1.0	2.3	13.3
Puerto Galera	Inside	2.0	0.0	2.7	0.0	0.4	9.9	0.1	15.1
	Outside	2.1	0.0	0.1	0.0	0.6	1.1	0.1	4.0
El Nido	Inside	5.5	0.0	1.9	0.4	0.2	14.9	1.5	24.4
	Outside	4.8	0.0	1.0	2.8	1.1	6.1	0.7	16.6
Dipaculao	Inside	2.9	1.6	0.0	0.1	0.0	0.9	2.1	7.5
	Outside	7.5	1.3	0.0	1.7	0.1	5.1	0.9	16.6
San Luis	Inside	2.3	0.0	0.0	0.0	0.1	1.0	0.1	3.5
	Outside	2.2	0.0	0.0	0.0	0.0	0.3	0.3	2.8
Siruma	Inside	3.3	1.0	0.3	4.8	0.6	7.1	6.4	23.5
	Outside	0.5	0.1	0.1	12.4	0.0	4.4	0.6	18.1
Tinambac	Inside	10.2	0.6	0.0	0.9	0.0	9.3	1.6	22.7
	Outside	0.0	0.3	0.0	0.4	0.0	5.5	0.8	7.0
Tigaon	Inside	2.8	1.0	0.1	11.0	0.2	0.1	0.7	15.9
	Outside	0.5	0.6	0.0	6.1	0.0	0.2	0.2	7.6
Sagnay	Inside	1.6	0.0	0.0	0.0	0.0	1.0	0.0	2.7
	Outside	13.9	0.1	0.0	0.1	0.1	0.6	0.1	14.8
Caramoan	Inside	1.3	0.9	0.0	1.0	0.0	2.8	1.4	7.3
	Outside	0.1	0.1	0.0	0.1	0.0	1.9	0.1	2.4
Bacacay	Inside	2.5	0.4	0.0	0.0	0.0	0.7	0.0	3.7
	Outside	1.7	0.0	0.0	0.0	0.0	0.7	0.0	2.5
Gubat	Inside	3.7	0.1	0.0	0.0	0.0	3.2	0.1	7.1
	Outside	4.5	0.1	0.0	0.1	0.1	2.2	0.0	6.9
Matnog	Inside	0.6	0.0	0.0	0.1	0.0	0.2	0.0	0.9
	Outside	5.0	0.0	0.1	0.0	0.0	1.6	0.0	6.6
Hinunangan	Inside	1.7	0.1	0.0	0.0	0.0	3.3	0.2	5.3
	Outside	0.6	0.0	0.0	0.0	0.0	0.5	0.2	1.3
San Francisco, Leyte	Inside	0.5	0.0	0.0	0.5	0.6	2.6	1.4	5.6
	Outside	0.0	0.0	0.0	0.6	0.0	1.0	0.1	1.6
Cantilan	Inside	5.1	0.0	0.1	0.3	0.6	3.4	0.2	9.7
	Outside	1.6	0.0	0.4	0.1	0.1	0.8	0.4	3.4
Lanuza	Inside	1.4	0.0	0.4	0.1	0.1	1.7	0.0	3.7
	Outside	1.9	0.0	0.0	0.1	0.0	2.1	0.0	4.0
Cortes	Inside	5.6	0.0	0.1	0.2	0.5	1.3	0.0	7.8
	Outside	1.8	0.0	0.1	3.5	0.1	0.4	0.0	5.9
Lianga	Inside	0.6	0.0	0.1	0.3	0.1	1.6	0.0	2.6
	Outside	0.7	0.0	0.3	0.1	0.0	1.1	0.0	2.2
Marihatag	Inside	0.9	0.3	0.0	0.1	0.3	2.8	0.1	4.6
	Outside	0.2	0.0	0.0	0.0	0.1	0.4	0.0	0.7
Mati City	Inside	8.5	0.0	0.2	0.0	0.3	0.6	0.1	9.7
	Outside	2.7	0.0	0.1	0.0	0.0	0.6	0.1	3.6
Roxas	Inside	1.1	0.2	0.1	0.9	0.3	0.9	0.4	3.9
	Outside	1.6	0.8	0.3	1.0	0.9	3.0	0.6	8.1
Bulalacao	Inside	0.2	0.0	0.0	0.0	0.0	2.6	0.1	3.0
	Outside	0.1	0.1	0.0	0.0	0.2	2.4	0.1	2.8
Siaton	Inside	10.5	0.0	0.4	0.4	2.4	0.7	2.7	17.2
	Outside	3.7	0.0	0.0	0.1	1.6	1.3	1.2	7.9
Bongao	Inside	10.5	0.3	0.0	0.0	0.3	4.1	1.0	16.3
	Outside	5.9	7.3	2.7	2.6	0.5	1.6	1.1	21.8
Panglima Sugala	Inside	6.3	10.8	0.1	3.8	0.7	9.0	0.6	31.3
	Outside	3.6	1.6	0.1	0.2	0.5	3.4	0.3	9.7
Simunul	Inside	17.0	1.0	0.8	0.4	0.6	4.6	1.0	25.5
	Outside	17.2	0.3	0.6	0.2	0.2	3.5	0.7	22.8
Calapan City	Inside	8.3	0.0	0.0	0.0	0.1	2.3	0.1	10.7
	Outside	2.3	0.0	0.0	0.0	0.7	0.0	0.0	3.0
Naujan	Inside	2.7	0.0	0.0	0.0	0.3	9.8	0.1	12.8
	Outside	0.7	0.0	0.0	0.1	0.1	0.1	0.0	1.0
Pola	Inside	3.3	1.8	0.1	0.8	0.9	4.3	0.2	11.3
	Outside	4.3	0.0	0.0	0.2	0.1	1.5	0.0	6.2
Gloria	Inside	10.0	16.9	0.2	6.1	1.7	7.2	0.8	43.0
	Outside	1.6	0.5	0.0	0.1	0.2	0.3	0.3	3.0
Bongabong	Inside	1.8	0.0	0.0	0.1	0.0	0.0	0.0	1.9
	Outside	0.2	0.0	0.0	0.0	0.0	0.0	0.0	0.3
Mansalay	Inside	0.5	0.0	0.0	0.0	0.1	1.9	0.1	2.6
	Outside	0.6	0.0	0.0	0.0	0.2	0.2	0.0	0.9
Bulan	Inside	0.0	0.1	0.0	0.0	0.1	1.7	0.2	2.1
	Outside	0.0	0.1	0.0	0.0	0.1	0.3	0.1	0.6
Ayungon	Inside	8.1	0.0	0.0	1.3	0.5	8.0	0.6	18.4
	Outside	2.0	0.0	0.0	0.2	0.1	0.4	0.5	3.2
Bindoy	Inside	4.7	0.0	0.0	0.2	0.2	4.2	0.7	10.1
	Outside	7.5	0.0	0.0	0.0	0.2	1.1	0.2	9.0
Amlan	Inside	4.5	2.3	0.0	11.4	0.7	7.6	1.9	28.4
	Outside	1.2	0.0	0.0	0.0	0.4	0.5	0.0	2.2
San Francisco, Cebu	Inside	12.7	0.5	0.0	3.3	2.5	5.0	0.2	24.2
	Outside	3.9	0.0	0.1	0.1	0.8	0.7	0.1	5.7
Pilar	Inside	4.3	0.0	0.0	0.6	0.2	0.6	0.2	5.9
	Outside	1.2	0.0	0.0	0.2	0.2	0.6	0.2	2.4
Boljoon	Inside	2.7	0.0	0.4	0.9	0.3	6.1	0.7	11.1
	Outside	4.2	0.0	0.0	0.2	1.0	6.9	0.3	12.6
Ubay	Inside	0.1	0.0	0.0	0.6	0.1	1.0	0.3	2.2
	Outside	0.0	0.0	0.0	0.0	0.1	0.5	0.2	0.8
Inabanga	Inside	0.1	0.2	0.0	0.5	0.2	10.5	2.4	13.9
	Outside	0.0	0.4	0.0	0.0	0.2	0.1	0.1	0.8
Tagbilaran	Inside	4.9	0.0	0.2	1.8	0.5	4.2	0.5	12.1
	Outside	0.0	0.0	0.0	0.0	0.0	1.3	1.2	2.6
Panabo City	Inside	0.1	0.0	0.0	0.0	0.0	0.2	0.0	0.3
	Outside	0.1	0.0	0.0	0.0	0.0	0.1	0.0	0.2
Dapia, IGACOS	Inside	7.8	0.0	0.0	0.0	0.0	1.2	0.0	9.1
	Outside	6.2	0.0	0.0	0.1	0.0	1.2	0.0	7.6
Sanipaan, IGACOS	Inside	10.4	0.5	0.0	0.0	0.0	0.7	0.1	11.7
	Outside	5.1	0.1	0.0	0.0	0.0	0.2	0.0	5.5
Dumalinao	Inside	1.2	0.4	0.0	0.0	1.3	5.0	0.2	8.1
	Outside	0.9	1.3	0.0	0.2	0.1	0.2	0.1	2.7
Tabina	Inside	7.0	0.6	2.2	2.2	1.6	7.4	2.5	23.6
	Outside	5.7	0.0	0.0	0.3	0.4	6.9	0.1	13.4
Tukuran	Inside	1.1	0.0	0.2	5.8	0.7	1.8	0.1	9.7
	Outside	0.7	0.0	0.0	0.1	0.1	0.5	0.2	1.5
Ipil	Inside	0.8	0.3	0.0	2.0	0.0	1.7	0.7	5.5
	Outside	0.2	0.0	0.0	3.8	0.3	1.0	0.2	5.4
TRNMP, Cagayancillo	Inside	26.8	2.7	4.3	8.4	0.8	6.4	5.8	55.1

## 2 Experimental design, materials, and methods

A modified non-destructive fish visual census (FVC) [2] was conducted on 4–12 10 m × 50 m belt transects for the 57 locally managed MPAs, half of which were established inside MPAs and the other half on adjacent reefs outside MPAs, which were at least 200 m away from the boundaries of MPAs. Thirty-three transects with the same dimension were surveyed for the nationally managed TRNMP. The transects were established on upper reef slope, mostly with depths ranging from 5 to 10 m. This was done by searching first for the reef crests then randomly laying the transects along an isobath. Surveys were conducted about 10–20 minutes after laying the transect to provide time for the disturbed fish to settle. FVC was performed by swimming slowly and stopping every 5 m to record all the fish within a 10 m - wide belt. Surveys were conducted by six professional divers who have been actively performing FVC surveys in the Philippines for more than a decade already. All surveys were conducted from 2006 to 2014 between 9:00–16:00 hours.

All the fish were counted, identified to the species level and total length (TL) estimated to the nearest centimeter. Some fishes were identifiable to genus level only. Fish biomass was estimated using the relationship between length (L) and weight (W) with the equation W = aL^b^. Species specific *a* and *b* values were gathered from published records [3], [4]. The a and b values used for fishes identified to the genus level only were the average values for the genus.

Classification of fish was based on [5], wherein only the surgeonfish (family Acanthuridae excluding genus *Zebrasoma*), parrotfish (subfamily Scarinae, family Labridae), snapper (family Lutjanidae), grouper (subfamily Epinephelinae, family Serranidae), goatfish (family Mullidae), sweetlips (family Haemulidae) and emperor (family Lethrinidae) were considered as commercially important fishes. These fish families constitute the major coral reef demersal fish caught by small-scale fishers in the Philippines [6].

The date of establishment and size of MPAs were gathered from the local records of the respective LGU or management body. These data are shown in [1] and are also available in the MPA database which is maintained by the Marine Protected Area Support Network (MSN) based at the Marine Science Institute, University of the Philippines Diliman [7].
